# Proposition to Establish a Lectureship on Dental Surgery in the Medical Colleges

**Published:** 1851-05

**Authors:** E. B. Gardette


To the Editors of the Medical Examiner.

*Gentlemen,*—I beg to ask the favor of you to republish the following article, from the "American Journal of Medical Sciences," in the May No. of the "Medical Examiner," if practicable. The object of this request is to correct, as promptly as possible, some errors made in printing the original, and to give publicity to the explanatory note, which, contrary to my expectations, was omitted in the American Journal of Medical Sciences.

Very truly and respectfully yours, &c,

E. B. GARDETTE.

### *Proposition to establish a Lectureship on Dental Surgery in the Medical Colleges.*

The undersigned, a practicing Dentist of Philadelphia, begs leave respectfully to call the attention of the trustees and medical faculties of the medical schools of the United States to the propriety and advantages of establishing an adjunct professorship to the chair of surgery, in which the speciality of Dental Surgery may be taught to the medical student seeking knowledge in their institutions.

In making this suggestion, he indulges the confident belief that the existence of such a chair would be no less useful to those who may be compelled to practice some branch of Dental Surgery, as part of their duties in general surgery, than to the smaller number who may determine to embrace that speciality as their profession.

The undersigned would offer for your consideration some reflections that seem to render this proposal consistent, not only with the wants of the student of medicine and the public, but with the duty and the interests of the medical schools which may act upon this request in such form as seems most proper to their own judgment.

It will scarcely be assumed by any trustee, and still less by any member of a medical faculty, that the profession of the Dentist or its duties, are less important to mankind than any other of the specialities of surgery—whether of the oculist, the aurist, or the lithotomist; and therefore it is needless to consider such a question open for debate, or requiring argument in a country, especially, where the services of good dentists have been so widely and universally needed. It being granted, then, that the science itself is of equal importance with the other departments of surgery, it is an undeniable truth that, whilst all other specialities are taught and included in the professorships of medical schools generally, the principles and practice of dental surgery have received no attention, or certainly none that would permit the graduate of medicine to feel that he was qualified for the simplest duty of the dentist.

Such a state of things was perhaps an unavoidable evil when men could not be found who were competent to teach the principles of our science, and when there was need of a comparatively small number of dentists; and hence no such aid was then called for from the medical colleges. But at this time there would seem to be required about twenty-five hundred dentists, more or less, throughout the United States, which is sufficiently proved by the fact that about that number are believed to be engaged in the practice of the profession, after some fashion or other—good, bad and indifferent; and to supply an increasing demand for well-educated dental surgeons, as well as to raise the standard of that profession, would appear to be a matter of common interest.

It is in evidence of the need of such improvement, that within a few years, two or three colleges of dental surgery have been established in this country under the sanction of charters from the States in which they are located; and without examining into their history or their influence upon the profession of the dentist, it is, at least, a source of regret that they must be regarded as the offspring of your neglect, and their very existence a reproach upon medical institutions for their unjust and exclusive course toward the science of Dental Surgery. It would seem an act of inexplicable unkindness to have thus singled out the profession of Dentist from among the specialities of Surgery, and forced it into an attitude of independent self-protection; but a gentleman who fulfils the duties of both medical practitioner and Dentist with such distinguished abilities as to be an honor to both professions, has traced truthfully the origin of the neglect of which the Dentist complains. The undersigned quotes from a valedictory address to the graduating class of Baltimore College of Dental Surgery, March 1850, when speaking of the early periods of the existence of a Medical Faculty:—

"They condemned, without stint, a calling they knew not how to practice, and a practice they knew not how to improve. Such of the faculty as were learned in their profession were found always competent and fully prepared to be oculists, aurists, or lithotomists, or to devote themselves to any other branch of the profession which their interest, inclination, or talents might determine, *except* that of dental surgery. This branch seemed to require something more than medical knowledge; it required great mechanical skill—an education of the hand as well as of the head—a kind of education they had not received and knew not where to acquire, and yet affected to despise. The necessities of the community cried aloud to them for help—a help which they could not bestow. This drove many sufferers ta seek dental aid out of the medical profession, and to obtain that help which mechanical genius alone could supply. At this the profession seemed mortified and chagrined, and loudly mocked at those who dared to supply their delinquencies, and united as one man in deriding the uneducated dental mechanic. They first created the necessity for an empiric, and then croaked forth their withering contempt on the creature their own ignorance had made."

Thus has dental surgery been left to struggle through endless impediments and difficulties, instead of being regarded as a link, however humble, in the great chain of medical science, which should with patriarchal strength and harmony foster and embrace every branch of the healing art.

It is now believed that, among the number of medical students who attend each of the schools, there are many who would gladly devote themselves to the speciality of dental surgery, and it is from among these, and at the early age at which they generally devote themselves to medical studies, that good Dentists could be formed; they should still be required to earn and receive a diploma with the title of M. D., as a guarantee of fitness to practice and a claim to confidence as dentists. Even among the number that annually graduate, either to practice medicine generally, or limiting their attendance to their own families and dependents at the South, a correct knowledge of the theory and practice of dental surgery, such as might be derived from a course of lectures, would be highly valuable. And it is believed there would not only be a direct increase of good Dentists, but a desirable addition to the number of those who should be competent to determine what constitutes a good dentist.

The establishment of separate schools for each speciality in medical science (and why should there not be for the Oculist, or the Aurist, as well as the Dentist?) would appear to be the dismembering of a great family, thereby lessening its influence for good, as well as that power which is justly derived from an *esprit de corps* subsisting in every branch of an elevated and honorable profession. In this expression of opinion as to the ultimate tendency of dental colleges, the undersigned is actuated by no unkind, selfish or invidious feelings, but, on the contrary, he gives due credit for the efforts that have been made and are making to improve the profession; and to the Baltimore College of Dental Surgery he is personally grateful for acts of courtesy and kindness towards himself, and to the memory of his father. In these Institutions it appears that the majority of Professors are medical men, and in some instances, teachers also in medical colleges, thus performing double duty. Would it not seem more consistent and reasonable to attach *one Dentist*, in the capacity of teacher, to a *Medical* Faculty, than to get up a *Dental* Faculty chiefly composed of *Medical men?*

The Dentist must be the teacher of Dentistry, and his profession is but an atom of Medical Science: it is in vain for him to attempt independence, or play the part of King Canute's flatterers to medical men: the tide of improvement in Dental Surgery thus far with all respect and due deference) is not under the control of the Doctors.

The educated dentists in the various parts of the United States are sufficiently numerous, it is believed, to fulfil such duties as might be assigned them in each of our medical colleges. The students who now attend the dental schools would in this event be added to the number of matriculants in the medical classes of the country, and the need of separate institutions would cease.

Frequent occasion for consultations between the physician, the surgeon, and the dentist, has been felt by almost every practitioner of medicine, as a necessary consequence of the connection and sympathy reciprocally existing between the teeth and many serious disturbances of the general health: such sympathies would naturally seem to suggest the mutual dependence between the physician, the surgeon, and the dentist, and should be a good reason for some similarity and sympathy in their modes of education. The benefits from such a state of things as the mind can readily anticipate would not rest here; medical men would sometimes, no doubt, receive important aid from the dentist, in tracing to their true origin many diseases of the head and face that now baffle their skill.

Whatever action your medical faculty or board of trustees may see fit to take in reference to these suggestions, and the object they are designed to promote, the undersigned may at least respectfully urge that they would seem to demand fair and kindly consideration, as involving matters of deep concern to the community, for whose safety and advantage all medical learning is sought, and medical colleges instituted and fostered.

E. B. Gardette.

Note.—It may be proper to state that this paper had been some weeks in the hands of the editor of the American Journal of Medical Sciences, for publication, when the writer was called upon to sign a petition to the Legislature of Pennsylvania for the establishment of a College of Dental Surgery, to be located in Philadelphia. This application for his name being the first knowledge he had of the existence of any such scheme, he could not join in its objects consistently with the opinions already promulgated. And besides, he has learned that a charter for a Dental College had been already obtained from the previous Legislature by Dr. J. R. Burden, who has not put it in active operation from the difficulty of finding suitable Dentists to fill the chairs. E. B. G.

